# The Transporter Classification Database (TCDB): 2021 update

**DOI:** 10.1093/nar/gkaa1004

**Published:** 2020-11-10

**Authors:** Milton H Saier, Vamsee S Reddy, Gabriel Moreno-Hagelsieb, Kevin J Hendargo, Yichi Zhang, Vasu Iddamsetty, Katie Jing Kay Lam, Nuo Tian, Steven Russum, Jianing Wang, Arturo Medrano-Soto

**Affiliations:** Department of Molecular Biology, Division of Biological Sciences, University of California at San Diego, La Jolla, CA 92093-0116, USA; Department of Molecular Biology, Division of Biological Sciences, University of California at San Diego, La Jolla, CA 92093-0116, USA; Department of Biology, Wilfrid Laurier University, Waterloo, Ontario N2L 3C5, Canada; Department of Molecular Biology, Division of Biological Sciences, University of California at San Diego, La Jolla, CA 92093-0116, USA; Department of Molecular Biology, Division of Biological Sciences, University of California at San Diego, La Jolla, CA 92093-0116, USA; Department of Molecular Biology, Division of Biological Sciences, University of California at San Diego, La Jolla, CA 92093-0116, USA; Department of Molecular Biology, Division of Biological Sciences, University of California at San Diego, La Jolla, CA 92093-0116, USA; Department of Molecular Biology, Division of Biological Sciences, University of California at San Diego, La Jolla, CA 92093-0116, USA; Department of Molecular Biology, Division of Biological Sciences, University of California at San Diego, La Jolla, CA 92093-0116, USA; Department of Molecular Biology, Division of Biological Sciences, University of California at San Diego, La Jolla, CA 92093-0116, USA; Department of Molecular Biology, Division of Biological Sciences, University of California at San Diego, La Jolla, CA 92093-0116, USA

## Abstract

The Transporter Classification Database (TCDB; tcdb.org) is a freely accessible reference resource, which provides functional, structural, mechanistic, medical and biotechnological information about transporters from organisms of all types. TCDB is the only transport protein classification database adopted by the International Union of Biochemistry and Molecular Biology (IUBMB) and now (October 1, 2020) consists of 20 653 proteins classified in 15 528 non-redundant transport systems with 1567 tabulated 3D structures, 18 336 reference citations describing 1536 transporter families, of which 26% are members of 82 recognized superfamilies. Overall, this is an increase of over 50% since the last published update of the database in 2016. This comprehensive update of the database contents and features include (i) adoption of a chemical ontology for substrates of transporters, (ii) inclusion of new superfamilies, (iii) a domain-based characterization of transporter families for the identification of new members as well as functional and evolutionary relationships between families, (iv) development of novel software to facilitate curation and use of the database, (v) addition of new subclasses of transport systems including 11 novel types of channels and 3 types of group translocators and (vi) the inclusion of many man-made (artificial) transmembrane pores/channels and carriers.

## INTRODUCTION

Membrane transporters constitute a diverse group of proteins that form intricate networks of channels, carriers, pumps, group translocators and electron flow carriers that determine the molecular compositions and energy status of cells (1). These proteins, which include ∼10% of all cellular proteins, transfer nutrients, end products of metabolism, toxic substances, macromolecules, signalling molecules, drugs, electrons, etc., from source to sink, resulting in the cellular uptake and extrusion of compounds and energy sources (2). Of particular importance to the fields of oncology, microbial pathogenesis and virology, drug efflux pumps play a dominant role in drug resistance both in pathogenic organisms and in cancer cells (3,4). Thousands of researchers worldwide contribute to the collective understanding of molecular transport across cellular membranes (5). TCDB (tcdb.org) is used by researchers over a million times per year involving about 100 000 different users.

In June 2001, the International Union of Biochemistry and Molecular Biology (IUBMB) formally adopted the Transporter Classification (TC) system as the only internationally recognized system for the organization of transport protein information derived from all organisms in the Earth's biosphere (6,7). With the advent of metagenomic sequencing and recent progress in computational biology (last 5 years), resulting in the discovery of dozens of novel phyla of nanobacteria and archaea, TCDB has expanded to include potential families of transporters found only in these novel phyla as well as proteins distantly related to members of pre-existing families. All transport systems are classified from evolutionary, structural and functional standpoints according to the original TCDB design (2,8). TCDB is not intended to be a collection of all transport-related protein sequences; instead, the ultimate goal is to identify and classify representative proteins of all transporter families in nature. Earlier versions of the TC database (TCDB) have been described in previous publications in Nucleic Acids Research (6,9–11).

As of now, TCDB provides access to data published in over 18 300 research papers. This information is integrated into descriptions and hierarchical structures within TCDB. The database now contains 15 528 single- or multi-component transport systems from all kinds of living organisms, and for 1567 of them, accessions for high resolution 3D structures in PDB (12) are available. These systems are classified into 1536 transporter families based on their phylogenies and functions. Many of these families have been found to be distantly related and are now classified into 82 superfamilies (13). This represents an overall growth of over 50% relative to our 2016 report (9). TCDB is continually updated as our software becomes more refined and as novel published research regarding transport systems becomes available. We hope these advances will increase the utility of the database for use by the international scientific community.

## RECENT DEVELOPMENTS

### Database expansion due to novel genomic and metagenomic analyses

Since the publication of our last update (9), we have carried out multiple genome and metagenome comparative analyses that have allowed the discovery of numerous novel transporter families derived from a variety of organismal types. Most, but not all, of these are of prokaryotic origin as progress in the discovery of novel phyla of bacteria and archaea has revolutionized our concept of the tree of life. We have carried out studies to identify transporters in many groups of pathogenic and probiotic bacteria. These include numerous pathogenic, symbiotic and free living species of spirochetes such as (a) *Leptospira* (14) and (b) *Treponema* (15), (c) species of *Bdellovibrio* that kill and eat other Gram-negative bacteria, either from within (intracellular) or from without (extracellular) the prey bacteria (16), (d) probiotic and pathogenic strains of *Escherichia coli* (17), and (e) probiotic and pathogenic *Bacteroides* species (18). In addition, we have ongoing projects on probiotic and pathogenic *Lactobacillus* species, seven species of sulfur reducing proteobacteria, four different phyla of the recently discovered ‘Candidatus Phylum Radiation’ (CPR) nanobacteria (19,20), and four phyla of the newly discovered Asgard archaeal superphylum, presumably the closest prokaryotic relatives of Eukarya (21). These analyses have resulted in 552 new proteins added to TCDB from metagenomes organized in 189 relatively small families. These proteins are representative of at least 100 000 non-redundant homologs in NCBI (BLAST *E*-value < 10^−6^, coverage >70%, and <90% sequence redundancy). From the 189 families with metagenomic members, 119 (62%) involve transporters of unknown mechanism of action (TC subclass 9.A) and putative transporters (TC subclass 9.B), including 17 families that seem to be exclusive to the newly discovered CPR and Asgard superphyla. Finally, the genomic analyses of some less well characterized eukaryotes such as red algae (22) as well as the plethora of experimental findings carried out by many research groups, worldwide, has allowed tremendous expansion of TCDB to include both well characterized and uncharacterized (putative) transport systems.

In our laboratory, transportome characterization requires the separate analysis of single- and multiple-component transport systems based on the knowledge available in TCDB. Furthermore, each newly sequenced (meta)genome provides the opportunity to identify potential transporters with little or no similarity to known transporters. Incorporation of these proteins into TCDB will continue to increase the coverage and sequence diversity in the database. We have developed a number of programs to tackle these challenges (see Table 1 and Supplementary File 1).

**Table 1.** Programs developed for this TCDB update. All programs running in the UNIX shell can be downloaded from the laboratory's software repository (https://github.com/SaierLaboratory)

Program^a,b,c^	Description
*Length Statistics Tool*	Plots the distribution of protein lengths across any TC class, superfamily or family (tcdb.org/progs/?tool=lens).
*Disease Explorer Tool*	Searches TCDB for disease-related transport systems (tcdb.org/progs/index.php?tool=disease).
*Re-entrant Loop Finder Tool*	Identifies re-entrant P-loops within transporters. This online tool is available in every page describing transport systems in TCDB.
*getSubstrates*	Generates a tab-delimited table with the ChEBI ID and names of the substrates annotated for all systems in TCDB (tcdb.org/cgi-bin/substrates/getSubstrates.py).
*listSuperfamilies*	Generates a tab-delimited table with the superfamily assigned to each system, subfamily and family in TCDB (tcdb.org/cgi-bin/substrates/listSuperfamilies.py).
*mkProteinClusters*	Generates a protein similarity tree based on BLAST or Smith-Waterman bit scores (30).
*extractFamily*	Downloads protein sequences from TCDB in various formats (30).
*famXpander*	Extracts non-redundant homologs from NCBI for a given TCDB family (30).
*areFamiliesHomologous*	Applies the transitivity property of homology to test whether two families are homologous and if they can be expanded into a superfamily (32).
*quod*	Generates highly customizable hydropathy plots (32).
*hvordan*	Generates graphical reports for each significant hit produced by areFamiliesHomologous (32).
*getDomainTopology*	Identifies conserved Pfam domains within a family of transporters (32).
*tmsRepeat*	Identifies repeat units of TMSs in transporter sequences (32).
*deuterocol*	Identifies homology between families by comparing bundles of transmembranal α-helices in 3D structures (32).
*findDistFamilyMembers*	Scans NCBI for distant members of a given TCDB family.
*singEasy*	Extracts all hits with single component systems in TCDB from *GBlast* results and classifies them into two groups, based on the reliability of the matches.
*getMultiCompSystems*	Exhaustive search of full genomes for matches against multicomponent systems in TCDB. This provides the raw data for programs that decide which systems are complete.
*YutanpaNet*	Network-based approach to infer complete multicomponent systems present in (meta)genomes based on the output of *getMultiCompSystems*.
*showSubnet*	Given the output of *YutanpaNet*, it generates a web-based graphical layout of the subnetwork for an input list of user-specified multicomponent systems.
*searchMissComponents*	Downloads homologs from NCBI matching user-specified functional keywords of missing target components and compares them against the query genome.
searchPseudogenes	Searches for missing components at the DNA level in the query genome (i.e. sequencing errors or pseudogenes).
*getOrthologs*	Compares full proteomes and infers orthologs as reciprocal best hits.
*matchDomains*	Compares protein sequences against databases of domains (i.e. Pfam, CDD, TIGRFAMs).
*cleanDomains*	Parses and filters the results of program *matchDomains*
*findNovelTrasporters*	Identifies potential transporters within a genome that show little or no sequence similarity to any transporter in TCDB.
*prepNewIMPs4TCDBupload*	Extracts non-redundant homologs for each protein identified by *findNovelTrasporters* in preparation for upload to TCDB.

^a^Four additional programs developed during the previous TCDB update were also extended and optimized (i.e. *GBlast*, *tmsplit*, *AveHAS*, *WHAT*).

^b^Programs were developed by Kevin Hendargo, Pranav Iddamsetty, Yichi Zhang, Vamsee Reddy, Gabriel Moreno-Hagelsieb and Arturo Medrano-Soto.

^c^For more details on each program see Supplementary File 1.

### Family and superfamily expansion

A major effort in our bioinformatics laboratory has been to develop software that allows the identification of distant relationships between families with the concomitant creation of superfamilies (Table 1 and Supplementary File 1). In TCDB, the operational definitions of family and superfamily have become more robust over time following the development of bioinformatics. A family is defined as a set of homologous proteins with common sequence, structural and functional attributes (i.e., topology, domains, motifs, folds, etc.). On the other hand, a superfamily is a group of distantly related families that may exhibit topological and functional differences, but interfamily comparisons reveal lower (but reliable) levels of sequence and structural similarities. Superfamilies can thus be created even when no single member has been functionally characterized. We have identified many new superfamilies and have expanded others that we had previously identified.

We had previously defined the LysE Superfamily with three families (23), but new work has revealed that it includes eleven families (24), each of which is specific for a different class of compounds. For example, the LysE family (TC# 2.A.75) exports basic amino acids while the RhtB family (TC# 2.A.76) exports small neutral amino acids, and the NAAT family (TC# 2.A.95) takes up neutral amino acids. However, four newly identified LysE superfamily families include members that transport a variety of inorganic ions: Cd^2+^ (CadD family; TC# 2.A.77), Mn^2+^ (Mtp family; TC# 2.A.107), Fe^2+^/Pb^2+^ (ILT family; TC# 2.A.108), or Ni^2+^/Co^2+^ (family NicO; TC# 2.A.113), depending on the family. The GAP family (TC# 2.A.116) seems to be specific for peptidoglycolipids which are exported to the cell walls of mycobacterial species, although only one member has been characterized (25). But this family has members from a wide range of bacteria and archaea and will probably prove to export different lipid types and possibly drugs. Surprisingly, still another member of this superfamily is the well characterized DsbD family (TC# 5.A.1) that exports electron pairs from a cytoplasm electron donor to extracellular disulfide-containing proteins (26,27).

The Tetraspan Junctional Complex (4JC) Superfamily is almost exclusively eukaryotic, with only one of the 15 member families being derived from bacteria (28). These proteins are the principal constituents of tight and gap junctions in animals, but some of these families are represented only in fungi while others have representation in a broader group of eukaryotes including viruses. Some form simple one-constituent endomembrane channels rather than multiprotein junctions.

The Membrane Attack Complex/Perforin (MACPF) superfamily includes three families that are derived exclusively from one of the three domains of life or are found more broadly in more than one (29). They can be part of the immune-related complement system of animals that combat infections by pathogenic microbes, or they can be of bacterial origin and cause diseases in animals.

The Anoctamin Superfamily includes seven eukaryotic families of calcium-activated cation or anion channels as well as lipid scramblases (30). Members of the first five families include proteins that are functionally characterized, some of which can both transport ions and scramble lipids, but the last two families have not yet been characterized except from bioinformatic standpoints. Presumably, the proteins of this superfamily do not have a prokaryotic ancestor; they may have evolved in eukaryotes. Novel mechanistic inferences, based on structure, have recently been forthcoming (31).

The Transporter/Opsin/G protein-coupled receptor (TOG) Superfamily has recently been expanded to include twelve families, and surprisingly, not all of the member families are typical transporters (32). They include light-dependent integral membrane chaperone proteins, photoreceptors and G-protein receptors. However, some of the photoreceptors can be mutated to transport cations, and some have a central channel which is blocked to prevent transport. A number of different G-protein receptors have been shown to flip lipids from one side of a bilayer to the other, and another transports cholesterol through a pore-type mechanism (33–35).

In 1993, Marger and Saier (36) described the Major Facilitator Superfamily (MFS) which then included five previously recognized families. Since then, we have published several papers that have resulted in very substantial expansion of this superfamily, until now, it is the largest transporter superfamily known with over one hundred distinct families (37). It is even larger than the prominent ATP-binding Cassette (ABC) superfamily of uptake/efflux porters which is polyphyletic (38). Until recently, all MFS family members were believed to be transporters. They utilize the proton motive force, not ATP hydrolysis, to energize transport. It has been known for some time that a very few MFS carriers have gained receptor functions, either together with, or instead of, their transport functions. However, with more sensitive methods, we were able to identify new, more distantly related families that do not have known transport functions (37). These include family (a) Lysyl tRNA synthetase (LysS; TC# 9.B.111); (b) Lysyl phosphatidyl glycerol synthases (MprF; TC# 4.H.1), which had been reported to flip their lipid products from the inside to the outside of the cell (39), (c) eukaryotic cytochrome b_561_ (Cytb_561_; TC# 5.B.2), (d) a poorly characterized family of proteins somehow involved in fungal conidiation and conidia germination (CCGP; TC# 9.B.57), and (e) an uncharacterized family in which all members contain domains of unknown function (DUF1275; TC# 9.B.143). Not all of these proteins are homologous to known MFS members throughout their entire lengths, but they at least share the basic repeat unit of six α-helical transmembrane segments (TMS) of the MFS domain. These observations provide interesting clues as to how proteins of similar domain structures evolve to serve very different biochemical functions (37).

Holins are phage or prokaryotic proteins that form ‘holes’ in cytoplasmic membranes to allow uptake of protons or release of proteins, and even to facilitate phage particle release. There are 58 holin families in TCDB, and some of them are large with multiple subfamilies. We have identified seven holin superfamilies, each with up to seven families (40,41).

There is a total of 76 outer membrane porin families in TCDB, and they are from Gram-negative bacteria, actinobacteria and eukaryotic organelles. Fifty-six of the porin families (74%) fall into a single superfamily (OMPP-I), and while one such family derives members from eukaryotic organelles, a second family functions to transfer electrons across the bacterial envelope, and several have not been functionally characterized. All others transport solutes either selectively or non-specifically. The other four superfamilies (OMPP-II–V) include only two families each (42). Two of these last four superfamilies (OMPP-II and V) have members derived only from actinobacteria while the other two are exclusively from plastids (OMPP-III) or from various eukaryotic organelles (plastids, mitochondria and peroxisomes) (OMPP-IV).

### A substrate ontology for TCDB

A recent addition to our transporter database is the comprehensive annotation of transported substrates based on the ontology of Chemical Entities of Biological Interest (ChEBI) (43). This is a formal representation of the properties of chemical entities and their relationships, thus allowing the organization of raw data into information and knowledge. As a result, each known substrate molecule or type is now associated with a ChEBI ID. Prior to this improvement, it was an arduous task to extract all transporters that utilized a carbohydrate substrate, for example. It would have also been impossible for third party software to organize and access our database according to chemical type.

We have completed a semi-automatic process of annotating over 7800 individual transport systems with a ChEBI ID that corresponds to the most selective substrates a system is known to transport. This process required natural language processing to extract chemical names from text descriptions and were all manually curated for accuracy. When new transport systems are added to TCDB, the ChEBI IDs of the corresponding substrates (if available) are now manually annotated at the time of entry. The advantage of adopting the ChEBI ontology is that each ChEBI ID exists within a hierarchy of terms with ‘parents’, ‘children’ and ‘synonyms’. The parent–children relationship is illustrated by the following example: ‘glucose’, ‘fructose’ and ‘galactose’ are ‘hexoses’, which are ‘carbohydrates’. Therefore, a search for ‘hexose’ will retrieve everything down the relational tree (i.e. glucose, fructose, galactose, etc.).

Substrate annotations for systems in TCDB are now available for download in tabular format via a webservice (see Table 1), which can be easily integrated by third party software. This opens the doors to many potential future applications. Substrate annotations can be used for genomic analysis, functional predictions, family/superfamily characterizations, and more. For example, genomes can be studied by observing the distribution of the types of transport systems present. A distribution of imported/exported substrates or substrate-types adds a valuable dimension to the analysis. A substrate-annotated genome can even benefit metabolic modelling projects. The ability to retrieve proteins that transport a particular substrate or substrate class may allow for improved methods for the prediction of molecular function and drug targets. Common motifs may be identified and associated with substrates, or statistical models such as hidden Markov models may be trained to predict substrates of unknown transporters. Another benefit of adopting the ChEBI ontology is that users can develop their own software tools to mine TCDB information while taking advantage of the application program interface provided by ChEBI to navigate the ontology (44).

The substrate ontology system fulfills a long-standing need in the transport biology and bioinformatics community. To the best of our knowledge, this is the first comprehensive and manually curated database of transport systems that standardizes substrate annotations by adopting a chemical ontology. In the future we plan to expand this ontology to include system modulators (i.e. activators, inhibitors etc.).

### Domain characterization of TCDB families (tcDoms)

We have started to produce tcDoms (Transporter Classification Domains), a set of Hidden Markov Models derived from families in TCDB. These tcDoms will be a useful resource (a) to sort query proteins into their corresponding TCDB families, (b) to infer relationships between families, and thus characterize superfamilies, or, when applicable, (c) to infer the substrates and functions of putative transporters. Although many transport-related domains are currently available in Pfam (45), CDD (46) and other databases, tcDoms are specialized in cellular transport, and are designed to assist in database manual curation, as well as to increase the robustness of family definitions in TCDB. In the initial phase, we are focusing on families composed exclusively of single component systems. In our approach, we first performed an all vs all comparison of all proteins in TCDB using BLAST (47) and selected those families where all protein members found each other. We then produced multiple alignments for these proteins using MUSCLE (48). Next, the program hmmbuild, from the HMMER software suite (49) was used to produce hidden Markov models from the multiple alignments. Finally, the performance of the resulting models was benchmarked using a leave-one-out cross-validation and checking against cross-contamination with unrelated families (manuscript in preparation). Of the 364 tcDoms currently built for 277 families, 295 overlap with 382 known Pfam models, and metagenomic proteins contribute to 66 (24%) of the families with tcDoms. Note that a one-to-one relationship between Pfam and tcDoms cannot be expected because different regions of proteins in a TCDB family can match different, nonoverlapping, Pfam domains, and different tcDoms can match the same Pfam domain. Our tcDoms are meant to help distinguish members of one family from those of another family, even if they belong to the same superfamily. Thus, we are expecting to produce more than one tcDom per family. The number of tcDoms per family depends on their relationships with other domain collections (e.g., Pfam and CDD), and the number of distinctive characteristic domains that we can identify for each family in TCDB. The initial set of hidden Markov models in tcDoms is available for download through the TCDB web site (tcdb.org/tcDoms.php).

### Software tools

Multiple programs have been developed and/or updated since 2016 to enhance the functionality of TCDB, mine the wealth of information stored therein, and support research projects that ultimately discover relevant data that can be added to TCDB. Table 1 presents a list of the programs developed for this update, and Supplementary File 1 gives details on each program. Most programs run in the UNIX shell, and a few are offered as web services.

## DISCUSSION

Since 2016, the number of representative transport systems and families in TCDB has grown by over 50%. This has been possible in part because of novel metagenomic data; 36% of the new families in this update include proteins from metagenomes. Moreover, combined with the improvement of software tools and developments in the field, TCDB now provides excellent opportunities to (a) explore relationships between families of transporters (5,13,28–32,37,40–42), (b) characterize and perform comparative analyses of transportomes encoded within genomes and metagenomes (14–18,22) and (c) derive inferences of function to be followed by experimental verification (50). The characterization of transporter families in TCDB, based on shared domains (tcDoms), allows rapid evaluation of family memberships for individual proteins, identification of evolutionary relationships, and inference of substrates and molecular functions. The adoption of the ChEBI ontology allows TCDB users to consider motif/domain relationships between non-homologous transporters with similar substrates, possibly identifying novel features that determine substrate recognition. The ontology can also be used to evaluate the distributions of substrates used not only within types of organisms, but also within the various families and superfamilies currently in TCDB. Since entirely new phyla of bacteria and archaea have been discovered within the last five years due to metagenomic analyses, the recently discovered sequence data have allowed the discovery of previously unrecognized putative transporter families unique to these newly discovered types of organisms. The new data also allow extension of relationships between organisms (i.e., archaea and eukaryotes). While monumental progress has been made in these regards, we are aware that the field is still young and rapidly expanding.

The organization and structure of TCDB allows us and other users to accommodate these additions and to ask more global questions than ever before. For example, what families in TCDB that do not belong to the same superfamily are potentially related or share regulatory domains? Of particular interest are transmembrane domains shared between established superfamilies, as they offer the possibility of grouping superfamilies at a higher level (e.g. ultra-superfamilies or super-superfamilies including two or more superfamilies). We have found evidence for such higher-order relationships, for example, between the family NicO (TC# 2.A.113), a member of the LysE superfamily, and the family NiCoT (TC# 2.A.52), a member of the TOG superfamily (32). Such relationships may arise because of addition to or subtraction from TMSs in a repeat unit. Similarly, we are interested in identifying multidomain proteins that link different superfamilies and investigate their impact on the evolution of families and their relationships. We are confident that TCDB will continue to be useful to the scientific community, and we always welcome suggestions for improvement.

## DATA AVAILABILITY

TCDB home page: tcdb.org. All software developed and maintained by the Saier lab for TCDB functionality and our research is available in our GitHub repository (github.com/SaierLaboratory).

## Supplementary Material

gkaa1004_Supplemental_File
