# Supplementary material for: The Transporter Classification Database (TCDB): 2021 update
Source: Nucleic Acids Res. 2020 Nov 10;49(D1):D461–7. doi: 10.1093/nar/gkaa1004 (PMC7778945; doi:10.1093/nar/gkaa1004)
Supplement: gkaa1004_Supplemental_File [file gkaa1004_supplemental_file.pdf]

Supplementary File 1

Software developed/updated for the 2021 TCDB update

*All scripts are available from our git repository: https://github.com/SaierLaboratory

---

Web services

This section presents online tools that have been developed to enhance the functionality of the TCDB server.

Length Statistics Tool

We created this tool to allow users to study the distribution of protein lengths across any TC class, superfamily or family. Results can be further sub-divided according to phylogenetic classifications to perform comparative statistical analyses. Currently this tool is only available online (tcdb.org/progs/?tool=lens).

Disease Explorer Tool

We created this tool to allow users to explore diseases associated with transport systems in TCDB. Users may search diseases by TC class, superfamily, or family. Users may also search specific diseases to identify the transporters associated with them. This tool is only available online (tcdb.org/progs/index.php?tool=disease).

Loop Finder Tool

We created this tool for identifying re-entrant loops (P-loops) within transporters in TCDB. This technique uses a combination of two methods to determine where re-entrant loops occur. An HMM model based on crystal structures of known re-entrant loops was initially used to find new examples of re-entrant loops in families where these loops were expected. These sequences were then used to create a BLAST database (1) of P-loops. The Loop Finder program uses a combination of HMMs and a special BLAST database to locate P-loops in proteins. This combined technique achieves higher sensitivity than either method alone. This tool is only available online in all TCDB pages describing systems.

Table of substrates for all systems in TCDB

We have added a service that generates a tab-delimited table of all available systems in TCDB and their most up to date annotated substrates, including their identifiers within the ChEBI ontology (2). This table will be useful to the community (tcdb.org/cgi-bin/substrates/getSubstrates.py).

Table of families/superfamilies for all systems in TCDB

We have added a service that generates a tab-delimited table with all systems in TCDB and their families and superfamilies. This parsable table allows the user to rapidly extract systems in specific families or superfamilies (tcdb.org/cgi-bin/substrates/listSuperfamilies.py).

WHAT

We have Improved the original program (3) by removing outdated JAVA dependencies. Hydropathy plots are now created using Python. Upgrades allow the program to run on newer systems and on most web servers. Added multiple parameters (e.g., what curves to display, resolution and format of the output image, labels, font size, etc.) that allow the user to customize the plots with publication quality (biotools.tcdb.org/barwhat2.html).

AveHAS

We have Improved the original program (4) by adding a feature to further analyze protein distributions according to amino acid types (branched, aliphatic, acidic, basic, etc.). As with our program *WHAT* (3), users are now able to specify what curves to display, the output format and resolution of the plots, as well as other graph parameters (e.g. grid, x-ticks spacing, font size for labels, etc.). Currently this program is only available online (biotools.tcdb.org/baravehas.html).

tmsplit

This program was improved to allow extracting specific regions of sequences, cutting a sequence into equal parts, cutting a sequence into groups of TMSs, or extracting a sequence region that covers a specific range of TMSs. We improved both the online (biotools.tcdb.org/bartms_split.html) and command line versions of the program.

Family and superfamily creation and expansion

This section describes tools that have been developed to aid in the identification of novel families of transporters, and the detection of distant relationships between existing families of transporters that can expand current or generate new superfamilies in TCDB.

mkProteinClusters

Given a number of proteins presumed to form a family or superfamily, this program performs hierarchical clustering based on their pairwise alignment bit scores and reports the results as a tree (5). The scores can be estimated using *BLAST* (6) or *SSEARCH* (7). Several methods for merging clusters are supported (i.e., Ward, single, average, complete and weighted).The clustering is performed using the R statistical

computing environment (r-project.org). This program is especially useful when working with large numbers of proteins, or with highly diverse proteins (as in a superfamily) where standard phylogenies cannot be constructed due to low-quality multiple alignments.

extractFamily

This program allows easy retrieval of protein sequences from TCDB (5). Sequences can be downloaded based on TCIDs at the subclass, family, subfamily and system levels, as well as the whole protein content in TCDB. The program can present the sequences in FASTA and 2-column formats (i.e., accession and sequence). In addition, it is possible to generate a BLAST database (1) with the specified sequences. Furthermore, sequences can be extracted from a local version of TCDB, which is useful when working with projects that freeze databases in a specific version.

famXpander

This program substitutes and extends the capabilities of *Protocol1* to retrieve homologs from NCBI (5). In addition to running *PSI-BLAST* (6) against the NCBI nonredundant database, retrieving large numbers of proteins and filtering results based on E-values and degrees of sequence redundancy, *famXpander* provides additional controls (i.e., minimal coverage of query and subject, minimal sequence lengths, retrieval of either the full sequences of subject proteins or just the aligned regions, and *BLAST* searches can be performed either locally or remotely using NCBI servers). This provides the raw data necessary to infer relationships between families, identify repeat units within a family and investigate conserved motifs and domains.

areFamiliesHomologous

Our programs *Protocol1* (8), *famXpander* (5), *Protocol2* (8) and *GSAT* (8) were integrated into a pipeline that significantly speeds up the analysis of distant evolutionary relationships between families using the transitivity property of homology, thus eliminating the possibility of human errors (5). For every significant *Protocol2* hit, *GSAT* scores are calculated across the full homology transitivity path. These hits can then be explored for hydropathy overlap, repeat unit compatibility, Pfam (9) domains, motif conservation, and 3D structural similarities.

quod

This program is a command line interface to our program *WHAT* (3) that allows more fine-grained control of hydropathy plots (10). In addition to parameters such as image format and resolution (useful for generating publication quality figures), the user can a) specify different colors for curves as well as for the TMSs, b) add TMSs manually (useful for correcting TMS predictions by *HMMTOP* (11) and other programs), c) add delimiting bars and wedges to indicate other regions of interest within sequences (e.g.,

alignment with other sequences), and d) add boxes to the plot (useful for incorporating information on domains or motifs).

hvordan

This program generates graphical reports that allow rapid interpretation of the top results reported by our program areFamiliesHomologous (10). It provides full sequence hydropathy plots for all 4 proteins involved in the homology transitivity analysis (A→B→ C→D). Hydropathy plots also show the regions and Pfam domains (9) involved in the alignments A→B, B→C, and C→D. *BLAST* comparisons against TCDB are also provided for proteins B and C, allowing fast visualization of the significance of the A→B and C→D alignments. The user can rapidly evaluate whether the proteins B and C, involved in the *Protocol2* (8) hits, constitute evidence of homology between the families represented by proteins A and D.

getDomainTopology

This program obtains the Pfam domain (9) composition of a TCDB family or superfamily (10). It first runs *HMMSCAN* (12) on every protein sequence to be examined against the Pfam database. If not all proteins in the family have a direct hit with a Pfam domain, the fragments of the sequences with direct hits are extracted and aligned to the sequences without direct hits using the Smith-Waterman algorithm as implemented in *SSEARCH* (7). This is interpreted as projecting the domains of sequences with direct Pfam hits onto sequences without Pfam hits. The expectation is that all sequences in a family should share at least one domain or have domains that belong to the same Pfam clan. This can further be used to relate families based on the overlap of shared domains. The rationale for this program was published in two of our publications (5,13).

tmsRepeat

We developed *tmsRepeat* as a strategy to identify repeated regions of TMSs within a transporter sequence, allowing the incorporation of prior knowledge about the TMS topology and the expected size of the repeat unit (10). The program first cuts the sequences of query transporters into TMS-bundles of a specified length depending on the size of the repeat unit expected by the user. TMS-bundles are then aligned with *SSEARCH* (7), and the results are printed in an HTML report that includes the hydropathy plots of the proteins under study, the limits of the bundles aligned and the regions within the bundles involved in the alignments.

deuterocol

The increasing availability of high-resolution 3D structures for multiple transporter families now allows analogous analyses to *areFamiliesHomologous* using 3D structural alignments. This program retrieves all structures available in PDB (14) for multiple families, extracts α-TMSs from OPM (15) and PDBTM (16), cuts them into α-helix bundles (depending on the length of the known or expected TMS repeat unit

shared among families) and superposes the resulting structures (10). Results can be filtered by several criteria including RMSD values, length and coverage of each bundle in the alignments. An html report is generated which includes links that allow visualization of each alignment.

findDistantFamilyMembers

This program is used when we want to identify remote members of a family in TCDB in order to increase the coverage of sequence diversity within a family. It consists of BLASTing all members within a family against the NCBI NR database and selecting hits that show borderline similarity to other members in the family, while having no significant similarity to any other families in TCDB. Alignments should satisfy a minimal coverage and involve a minimal number of TMSs compatible with the topology of the family under analysis. This produces a list of candidates that should be further evaluated by an expert.

Comparative genomics

The programs in this section allow the characterization of transportomes of full genomes or metagenomes as well as their comparisons.

GBlast

This program is used for the characterization of transporters in (meta)genomes and is best suited for the identification of single-component systems (8). We improved it to allow additional sorting of results by TCID (as opposed to only by E-value). TCIDs are now hyperlinked to the corresponding entries in TCDB. We added columns describing the coverage of the query and subject in the alignments as well as the substrates reported in TCDB for each matched entry. In addition, hydropathy plots for the full-length query and subject proteins are now included, indicating with bars and wedges the regions of the proteins covered in the alignments.

singEasy

This program extracts from the *GBlast* (8) results all matches with single component transport systems in TCDB and classifies them into either confident (good E-values and alignment coverage) or suspects (poor E-values and/or poor alignment coverage). This is because *GBlast* includes matches with multicomponent systems without regard as to whether the full systems were found in the genome. Other programs focus on the analysis of multicomponent systems.

getMultiCompSystems

This program complements *GBlast* (8) and represents the first step in the characterization of multicomponent systems encoded within (meta)genomes. All multicomponent systems available in TCDB

are downloaded and BLASTed against the genome allowing users to specify the E-value cutoff, minimal coverages of the query and subject sequences, sequence identity, minimal alignment length, and more. Hydropathy plots are generated depicting the regions in the query and subject proteins that are aligned and covered by Pfam domains (9). The program also determines the genomic context of homologs of each of the components within a system. Given that in prokaryotes multicomponent systems are often found in operons, the genomic context facilitates the selection of components that are members of the same operon.

YutanpaNet

The output of *getMultiCompSystems* can be quite extensive and thus difficult to interpret. *YutanpaNet* tackles this challenge by applying a network-based approach to mine such a large data set and identify complete multicomponent systems within a query genome or metagenome. In our strategy, we use a weighted scoring function and a selection function to make the assignments. For each alignment, the program calculates a score that weighs a) the E-value; b) the alignment coverage; c) the presence of transmembrane vs hydrophilic domains; d) shared Pfam domains (9); e) shared genomic contexts between genes matching the same system, and f) identified protein fusions. *YutanpaNet* ranks all multicomponent systems identified in the genome and groups them into four categories: i) high confidence: complete systems where every component satisfies all criteria; ii) medium-high confidence: potentially complete systems but minor issues are observed, e.g., one component shows a good E-value and high coverage but does not have a Pfam match with the domain observed in TCDB; iii) medium-low confidence: systems may be complete if further research by the user indicates that missing components are not essential for function (e.g., accessory proteins), or that proteins with low coverage include the functionally relevant domains; and iv) low confidence: This would be remaining systems that could not be completed automatically because too many components are missing.

ShowSubnet

To verify the validity of assignments made by *YutanpaNet*, an efficient graphical visualization of the assignments is necessary. *ShowSubnet* uses the core network files generated by the program *YutanpaNet* to create an interactive graphical layout of any subnetwork involving user-specified systems in TCDB or proteins in the query genome. The network groups the systems and their components as annotated in TCDB and shows links to all proteins in the query genome matching the components. The colors of the edges indicate the coverage (good or low) of the alignments between proteins in the query genome and the corresponding components in TCDB; the widths of the edges are proportional to the quality of the alignments; the shapes of the nodes denote the type of objects (i.e., a system, a component or a protein in the query genome). The genomic context is conveyed via the spatial relationships between nodes representing genome proteins, that is, nodes located along a horizontal line are neighbors. Some subnetworks can be quite large (e.g. ABC systems), so the interface also includes a search function to

identify specific proteins. This tool helps the user visualize homologous systems competing for the same set of genes in the query genome and allows validation of the initial assignments by program *YutanpaNet*.

searchMissComponents

It is common that after running *getMultiComSystems* and *YutanpaNet*, there are components still missing in a transport system. This program takes functional keywords associated with the missing component and downloads from NCBI all non-redundant proteins containing those keywords in their annotations. Sequences are then BLASTed against the query (meta)genome, and for all significant hits, hydropathy plots and Pfam domains (9) are shown to help the user determine whether a given missing component was found. This is useful when the genome under study has remote homologs of the corresponding component in TCDB. The user must be careful in selecting the right functional keywords to avoid downloading proteins from NCBI unrelated to the missing component under analysis.

searchPseudogenes

When other strategies have failed to identify missing components in a transport system (see *searchMissComponents*), this program searches the DNA sequence of the query genome for the footprint of the missing component. This is to test the possibility that the missing components were not detected due to sequencing/annotation errors or because the corresponding genes turned into pseudogenes. This program is a wrapper for *BLASTX* (1) to compare the protein sequences of missing components against the DNA sequence of the query genome.

getOrthologs

This program compares full proteomes and infers pairs of orthologous genes based on the reciprocal best hit approach. The program can use any one of several programs to perform the sequence alignments (i.e., *BLAST* (6), *DIAMOND* (17), *LASTAL* (18) and *MMseqs* (19)). Various levels of sensitivity can be used when running *diamond*. Furthermore, the program offers options for controlling how a candidate protein fusion will be treated based on cutoffs for minimal alignment coverage and maximal E-value.

matchDomains

This program identifies domains in protein sequences based on several programs (i.e., *HMMSCAN* (12), *RPS-BLAST* (20) or *mmseqs* (19)) and domain databases (i.e., Pfam (9), CDD (21,22), COG (23), TIGRFAMs (24)). This program facilitates comparisons of domain contents between genomes and the identification of characteristic domains within families and superfamilies.

cleanDomains

This program parses the output files generated by *matchDomains* and extracts useful data based on user-defined minimal coverage of domain models and maximal overlap between domains.

findNovelTransporters

In transportome characterization, the main task is the identification of transporters for which there is knowledge available in TCDB. This program performs the other side of the analysis, that is, the identification of integral membrane proteins in the genome that are likely transporters (e.g., by having a minimal number of TMSs), but show no significant similarity to any protein in TCDB. The user can specify the minimal number of TMS in the expected transporters, the alignment coverage and the E-value cutoff. For each protein that passes these criteria the program reports hydropathy plots that can be used by curators to ultimately decide whether proteins are good candidates for novel families in TCDB.

prepNewIMPs4TCDBupload

After running *findNovelTransporters* and selecting the list of candidate novel integral membrane proteins in a reference genome that may potentially be transporters, this program extracts non-redundant homologs from NCBI that will be used by TCDB curators to create the new family.

References

1. Camacho, C., Coulouris, G., Avagyan, V., Ma, N., Papadopoulos, J., Bealer, K. and Madden, T.L. (2009) BLAST+: architecture and applications. *BMC Bioinformatics*, **10**, 421.
2. Hastings, J., Owen, G., Dekker, A., Ennis, M., Kale, N., Muthukrishnan, V., Turner, S., Swainston, N., Mendes, P. and Steinbeck, C. (2016) ChEBI in 2016: Improved services and an expanding collection of metabolites. *Nucleic Acids Res*, **44**, D1214-1219.
3. Zhai, Y. and Saier, M.H., Jr. (2001) A web-based program (WHAT) for the simultaneous prediction of hydropathy, amphipathicity, secondary structure and transmembrane topology for a single protein sequence. *J Mol Microbiol Biotechnol*, **3**, 501-502.
4. Zhai, Y. and Saier, M.H., Jr. (2001) A web-based program for the prediction of average hydropathy, average amphipathicity and average similarity of multiply aligned homologous proteins. *J Mol Microbiol Biotechnol*, **3**, 285-286.
5. Medrano-Soto, A., Moreno-Hagelsieb, G., McLaughlin, D., Ye, Z.S., Hendargo, K.J. and Saier, M.H., Jr. (2018) Bioinformatic characterization of the Anoctamin Superfamily of Ca2+-activated ion channels and lipid scramblases. *PLoS One*, **13**, e0192851.
6. Altschul, S.F., Madden, T.L., Schaffer, A.A., Zhang, J., Zhang, Z., Miller, W. and Lipman, D.J. (1997) Gapped BLAST and PSI-BLAST: a new generation of protein database search programs. *Nucleic Acids Res*, **25**, 3389-3402.
7. Pearson, W.R. (1991) Searching protein sequence libraries: comparison of the sensitivity and selectivity of the Smith-Waterman and FASTA algorithms. *Genomics*, **11**, 635-650.

8. Reddy, V.S. and Saier, M.H., Jr. (2012) BioV Suite--a collection of programs for the study of transport protein evolution. *FEBS J*, **279**, 2036-2046.

9. El-Gebali, S., Mistry, J., Bateman, A., Eddy, S.R., Luciani, A., Potter, S.C., Qureshi, M., Richardson, L.J., Salazar, G.A., Smart, A. *et al.* (2019) The Pfam protein families database in 2019. *Nucleic Acids Res*, **47**, D427-D432.

10. Medrano-Soto, A., Ghazi, F., Hendargo, K.J., Moreno-Hagelsieb, G., Myers, S. and Saier, M.H., Jr. (2020) Expansion of the Transporter-Opsin-G protein-coupled receptor superfamily with five new protein families. *PLoS One*, **15**, e0231085.

11. Tusnady, G.E. and Simon, I. (2001) The HMMTOP transmembrane topology prediction server. *Bioinformatics*, **17**, 849-850.

12. Eddy, S.R. (2011) Accelerated Profile HMM Searches. *PLoS Comput Biol*, **7**, e1002195.

13. Moreno-Hagelsieb, G., Vitug, B., Medrano-Soto, A. and Saier, M.H., Jr. (2017) The Membrane Attack Complex/Perforin Superfamily. *J Mol Microbiol Biotechnol*, **27**, 252-267.

14. Burley, S.K., Berman, H.M., Christie, C., Duarte, J.M., Feng, Z., Westbrook, J., Young, J. and Zardecki, C. (2018) RCSB Protein Data Bank: Sustaining a living digital data resource that enables breakthroughs in scientific research and biomedical education. *Protein Sci*, **27**, 316-330.

15. Lomize, M.A., Pogozheva, I.D., Joo, H., Mosberg, H.I. and Lomize, A.L. (2012) OPM database and PPM web server: resources for positioning of proteins in membranes. *Nucleic Acids Res*, **40**, D370-376.

16. Kozma, D., Simon, I. and Tusnady, G.E. (2013) PDBTM: Protein Data Bank of transmembrane proteins after 8 years. *Nucleic Acids Res*, **41**, D524-529.

17. Buchfink, B., Xie, C. and Huson, D.H. (2015) Fast and sensitive protein alignment using DIAMOND. *Nat Methods*, **12**, 59-60.

18. Frith, M.C. and Kawaguchi, R. (2015) Split-alignment of genomes finds orthologies more accurately. *Genome Biol*, **16**, 106.

19. Mirdita, M., Steinegger, M. and Soding, J. (2019) MMseqs2 desktop and local web server app for fast, interactive sequence searches. *Bioinformatics*, **35**, 2856-2858.

20. Marchler-Bauer, A., Panchenko, A.R., Shoemaker, B.A., Thiessen, P.A., Geer, L.Y. and Bryant, S.H. (2002) CDD: a database of conserved domain alignments with links to domain three-dimensional structure. *Nucleic Acids Res*, **30**, 281-283.

21. Yang, M., Derbyshire, M.K., Yamashita, R.A. and Marchler-Bauer, A. (2020) NCBI's Conserved Domain Database and Tools for Protein Domain Analysis. *Curr Protoc Bioinformatics*, **69**, e90.

22. Lu, S., Wang, J., Chitsaz, F., Derbyshire, M.K., Geer, R.C., Gonzales, N.R., Gwadz, M., Hurwitz, D.I., Marchler, G.H., Song, J.S. *et al.* (2020) CDD/SPARCLE: the conserved domain database in 2020. *Nucleic Acids Res*, **48**, D265-D268.

23. Galperin, M.Y., Kristensen, D.M., Makarova, K.S., Wolf, Y.I. and Koonin, E.V. (2019) Microbial genome analysis: the COG approach. *Brief Bioinform*, **20**, 1063-1070.

24. Haft, D.H., Selengut, J.D., Richter, R.A., Harkins, D., Basu, M.K. and Beck, E. (2013) TIGRFAMs and Genome Properties in 2013. *Nucleic Acids Res*, **41**, D387-395.
